# Prevalence and correlates of HIV, syphilis, and hepatitis B and C infection and harm reduction program use among male injecting drug users in Kabul, Afghanistan: A cross-sectional assessment

**DOI:** 10.1186/1477-7517-8-22

**Published:** 2011-08-25

**Authors:** Catherine S Todd, Abdul Nasir, M Raza Stanekzai, Katja Fiekert, M Zafar Rasuli, David Vlahov, Steffanie A Strathdee

**Affiliations:** 1 Department of Obstetrics & Gynecology, Columbia University, New York, New York, USA; 2 Health Protection and Research Organization, Kabul, Afghanistan; 3 United Nations Office of Drugs and Crime, Afghanistan Country Office, Kabul, Afghanistan; 4 Ministry of Counter Narcotics, Islamic Republic of Afghanistan, Kabul, Afghanistan; 5 Center for Urban Epidemiologic Studies, The New York Academy of Medicine, New York, NY, USA; 6 Division of Global Public Health, University of California, San Diego, La Jolla, CA, USA

**Keywords:** injection drug user, Afghanistan, HIV, hepatitis C, harm reduction

## Abstract

**Background:**

A nascent HIV epidemic and high prevalence of risky drug practices were detected among injecting drug users (IDUs) in Kabul, Afghanistan from 2005-2006. We assessed prevalence of HIV, hepatitis C virus (HCV), hepatitis B surface antigen (HBsAg), syphilis, and needle and syringe program (NSP) use among this population.

**Methods:**

IDUs were recruited between June, 2007 and March, 2009 and completed questionnaires and rapid testing for HIV, HCV, HBsAg, and syphilis; positive samples received confirmatory testing. Logistic regression was used to identify correlates of HIV, HCV, and current NSP use.

**Results:**

Of 483 participants, all were male and median age, age at first injection, and duration of injection were 28, 24, and 2.0 years, respectively. One-fifth (23.0%) had initiated injecting within the last year. Reported risky injecting practices included ever sharing needles/syringes (16.9%) or other injecting equipment (38.4%). Prevalence of HIV, HCV Ab, HBSAg, and syphilis was 2.1% (95% CI: 1.0-3.8), 36.1% (95% CI: 31.8-40.4), 4.6% (95% CI: 2.9-6.9), and 1.2% (95% CI: 0.5-2.7), respectively. HIV and HCV infection were both independently associated with sharing needles/syringes (AOR = 5.96, 95% CI: 1.58 - 22.38 and AOR = 2.33, 95% CI: 1.38 - 3.95, respectively). Approximately half (53.8%) of the participants were using NSP services at time of enrollment and 51.3% reported receiving syringes from NSPs in the last three months. Current NSP use was associated with initiating drug use with injecting (AOR = 2.58, 95% CI: 1.22 - 5.44), sharing injecting equipment in the last three months (AOR = 1.79, 95% CI: 1.16 - 2.77), prior incarceration (AOR = 1.57, 95% CI: 1.06 - 2.32), and greater daily frequency of injecting (AOR = 1.40 injections daily, 95% CI: 1.08 - 1.82).

**Conclusions:**

HIV and HCV prevalence appear stable among Kabul IDUs, though the substantial number having recently initiated injecting raises concern that transmission risk may increase over time. Harm reduction programming appears to be reaching high-risk drug user populations; however, monitoring is warranted to determine efficacy of prevention programming in this dynamic environment.

## Introduction

Central Asia has been identified as a region of concern for expanding human immunodeficiency virus (HIV) epidemics largely driven by injecting drug use [1,2]. Increased rates of drug use, particularly injecting use, in this region have been attributed to multiple factors, including high unemployment, political instability, and, predominantly, an increasing opiate supply from Afghanistan [2,3]. Though Afghanistan's role as an opium producer is widely known, consumption within the country has received relatively less attention [4,5]. Based on a 2009 national survey, approximately 2.7% of the adult population is estimated to use opiates, and both smoking and injection of heroin are believed to be increasing [5]. The majority of drug use is concentrated in urban areas, particularly injecting use, where 20% of urban heroin users are estimated to inject [5]. A 2007 mapping study estimated a total injecting drug user (IDU) population of 1465 across three Afghan cities, with 1261 in Kabul city [6].

In 2005-2006, we conducted a cross-sectional assessment of the seroprevalence of HIV and viral hepatitis among 464 Kabul IDUs [7]. Although HIV prevalence was low at 3.1%, HCV prevalence was higher at 36%. Levels of lifetime risk behaviors were high, reflected by 50.4% having ever shared needles or syringes, and harm reduction program use was low, with only one participant reporting needle and syringe collection and distribution program (NSP) use [7,8]. Since the 2006 study, drug use patterns and the number of drug users have changed, with the numbers of drug users, injectors, and those using heroin as their drug of choice increasing in 2009 [5]. In this interval, the number and range of harm reduction services have also expanded, particularly in Kabul. In 2009, HIV prevalence among IDUs in Kabul was 3%, suggesting some stability in the environment since 2006 [7,9]. This biobehavioral survey also noted that 98% of Kabul IDUs reported using a sterile needle at their last injection; however, the national drug use survey performed in 2009 indicated that 87% of IDUs reported lifetime needle sharing and that 60% reported their syringes had been used by between 2 and 5 people prior to their personal use [5,9]. These data appear contradictory and clarification of injecting behaviors is needed, for which serial prevalence data may provide some objective measures.

The purpose of this manuscript is to describe the prevalence and correlates of HIV, syphilis, and hepatitis B and C and harm reduction program use among IDUs entering a cohort recruited in Kabul, Afghanistan between June, 2007 and March, 2009.

## Methods

### Setting

Kabul is the capital and largest city in Afghanistan, estimated to have a population of 2.8 million in 2008 [10]. Kabul also has among the highest density of opium and heroin users and IDUs nationally [5]. At the beginning of the recruitment period, three NSPs were operating as were two addiction treatment programs available at no cost. No methadone maintenance programs were operating during the enrollment period.

### Participant Eligibility & Recruitment

IDUs aged 18 years or greater who had injected within the last 30 days and residing in Kabul were eligible for entry. Potential participants were recruited at sites within Kabul city frequented by drug users and through NSPs by trained study staff; no set sample size was employed for recruitment from either site. Recruitment at sites frequented by drug users was done as a variant on time-location sampling, as study staff were present for enrollment at each of four to five sites based on the number of potential participants present at those sites. Site selection was re-evaluated monthly as congregation sites frequently shifted for various reasons, such as police harassment. Further, study staff would regularly visit pharmacies throughout the city and inquire with harm reduction outreach workers and participants regarding new sites for IDU congregation. Sites were distributed throughout Kabul, but, for the majority of the enrollment period, were largely concentrated in the western sections of the city, consistent with reported and confirmed IDU presence throughout the study period. Potential participants were also approached at drop-in centers for the three harm reduction programs in Kabul; study staff enrolled or performed follow-up interviews for participants recruited at these sites one afternoon each week throughout the study. For this convenience sample, IDUs interested in participation accompanied staff to a private room at the harm reduction center or to the study office for explanation of the study and provision of informed consent. Due to low literacy rates, potential participants were asked a series of questions following presentation of the consent by study staff to ensure comprehension; fingerprint was provided in lieu of signature per participant preference. Potential participants declining entry were not enumerated nor was any information collected from these individuals. The institutional review boards of the Afghan Ministry of Public Health, University of California San Diego, and Columbia University approved the protocol.

### Measurement of variables and outcomes of interest

The questionnaire instrument assessed sociodemographics, travel, incarceration and medical histories, drug use and sexual behaviors, and knowledge of infection transmission and prevention. Drug use behaviors of interest included sharing needles/syringes ever and in the last three months, sharing injecting "works" (e.g. cookers, cotton) ever and in the last three months, duration of injecting, injecting while incarcerated, aspirating and re-injecting blood (*khoon bozee*), and receiving assistance with injecting. Assistance with injecting was assessed with the question of whether anyone, including a group leader, had injected a given participant. Transition from smoking to injecting opiates or other substances was also specifically queried, as was geographic location of injecting initiation.

Sexual risk behaviors of interest were having used a condom at any sexual encounter, ever having sex with men or boys, and having patronized a female sex worker (FSW) ever and in the last three months. Other possible routes of transmission were also assessed, such as receipt of therapeutic injections.

The outcomes of interest were prevalence of HIV, syphilis, and hepatitis B and C infection and reported NSP program use. NSP program use at enrollment was defined as both current stated participation and reporting currently receiving syringes from a program.

### Procedures

Study staff were Afghan men who had worked with drug users through either harm reduction programs or through medical practice and included at least one physician at all times. Staff were trained in human subjects research, voluntary counseling and testing practices, phlebotomy and serum preparation, and questionnaire administration. The senior study managers throughout the project, both Afghan physician (MRS and AN), provided quality assurance in the field through instrument completion checks and direct observation.

Following informed consent, participants completed the interviewer-administered questionnaire, received pre-test counseling, and underwent whole blood rapid testing for HIV-1, syphilis, hepatitis B surface antigen (HBsAg), and hepatitis C antibody (HCV Ab) (all rapid tests from SD Bioline, Standard Diagnostics, Kyonggi-do, Korea). Participants with reactive rapid test results underwent intravenous sampling for confirmatory testing. Participants then received post-test counseling, hepatitis B vaccine as needed, and were scheduled for health education classes and follow-up tests results, as indicated. All participants completed four health education classes covering injection and sexual risk reduction, abscess prevention, and nutrition and hygiene. Basic hygiene supplies (e.g. soap, razor, toothbrush) were provided upon completion of the classes (value US$4), not advertised at the time of recruitment to avoid coercion.

### Laboratory Testing

All confirmatory testing was performed at the Afghan Public Health Institute laboratory in Kabul. Samples reactive for HIV on rapid test received Western blot confirmation (HIV Blot 2.2, Genelabs, Singapore). For hepatitis C, polymerase chain reaction (PCR) (Amplicor, Roche Diagnostics, Mannheim, Germany) was used to confirm infection with reflex RIBA (recombinant immunoblot assay) (Chiron RIBA 3.0 SIA, Chiron Company, Emeryville, California) employed for samples with no detectable virus. HBV confirmation was performed with PCR (Amplicor, Roche Diagnostics, Mannheim, Germany) for reactive specimens. Syphilis infection was confirmed with *Treponema pallidum *plasma agglutinin assay (TPPA) (Fujirebio, Wilmington, DE, USA), with rapid plasma reagin (RPR) titre (Inverness Medical, Princeton, NJ, USA) for guidance of clinical decision-making. Participants were encouraged to follow up for confirmatory tests results but were not actively sought to preserve confidentiality.

Treatment referrals were provided for participants with confirmed cases of HIV, hepatitis B, and/or C receiving test results to sites within the public health system providing antiretroviral therapy and hepatitis supportive care. Confirmed syphilis cases with RPR titre≥1:4 were offered treatment with intramuscular benazathine penicillin per international treatment guidelines [11].

### Statistical Analysis

The target sample size for cohort entry was 450, sufficient to detect at least a 16% difference in needle sharing over time with 80% power (two-sided alpha = 0.05). Descriptive statistics for the study population were generated. Prevalence and confidence intervals for each infection were calculated with Poisson or binomial distribution as appropriate. Prevalence of NSP use at baseline was measured with simple proportions.

Univariable logistic regression was performed to identify potential associations between outcomes of interest and select demographic and risk behavior variables. Variables were entered into a multiple model if they were associated at the 10% level in univariable analysis or were considered of epidemiologic significance; entry into the final model was determined by likelihood ratio test at a significance level <0.10. All analysis was performed with Stata Version 10.0 (Stata Corporation, College Station, Texas).

## Results

### Participant and Drug Use Characteristics

There were 483 participants recruited into the cohort over a period of 18 months. Demographic and injecting risk behavior characteristics are reported in Table 1. Briefly, all participants were male and were living in Kabul, with most living in districts 3 (37.1%), 5 (11.7%), 13 (11.5%), 1 (10.2%), 6 (6.9%), 8 (6.3%), and 2 (6.0%) of the 17 city districts. Most were Afghan nationality and had lived outside Afghanistan in the last 5 years. The living situations of participants at time of cohort entry were living with immediate (40.2%) or extended (2.5%) family, other drug users (31.0%), or were homeless (22.5%).

**Table 1:** Demographic and key risk behavior characteristics of male injecting drug users enrolled into a longitudinal cohort study in Kabul, Afghanistan (N = 483)

Continuous Variables	Mean	Median	IQR
Age (years)	29.6	28	24-33

Level of education (years)	5.3	5	0-7

Age initiated injecting (years)	25.8	24	21 - 29

Duration of injecting (years)	4.0	2	1 - 6

Number of daily injections	5.7	6	5 - 6

Dichotomous Variables	N		%

Ever married	224		46.4

Born in Afghanistan	418		87.1

Lived outside Afghanistan in last 5 years	310		64.7

Employed at enrollment	56		11.7

Prior incarceration	302		63.1

Initiated drug use with injecting	39		8.1

Initiated injecting in the last year	111		23.0

Initiated injection inside Afghanistan	321		66.5

Ever shared needles/syringes	81		16.9

Shared needles/syringes in last 3 months	31		6.4

Ever shared injecting equipment	189		38.4

Shared injecting equipment in last 3 months	132		27.3

Ever inject/re-aspirate blood (*khoon bozee*)	337		70.2

Prior addiction treatment	70		14.6

Ever paid woman for sex*	177		40.4

Ever had sex with another male*	49		11.1

Ever used condom*	49		11.1

*Of 443 participants ever reporting previous sexual activity.

N = Number

IQR- Interquartile Range

Heroin mixed with avil (76.8%) or with water or lemon juice (22.3%) were the most frequent drug preparations reportedly used in the month prior to enrollment. Use of other pharmaceutical preparations for injection was exceedingly rare, as only five participants mentioned using benzodiazepines or phenobarbital in combination with heroin and no participants mentioned using pharmaceutical agents alone in the last month. Other injecting risk behaviors, including lifetime and recent needle/syringe and injecting equipment sharing and aspirating and re-injecting blood (*khoon bozee*), were common (Table 1). Generally, Afghanistan was the site for injecting initiation although about one-third initiated injecting as a refugee.

### Prevalence and Correlates of Infections

Prevalence of HIV, HCV Ab, HBV, and syphilis was 2.1% (CI: 1.0-3.8), 36.1% (CI: 31.8-40.4), 4.6% (CI: 2.9-6.9), and 1.2%,(CI: 0.5-2.7), respectively. Of those who were HIV-infected, 80% were co-infected with HCV. In logistic regression analysis, HIV infection was significantly associated with ever sharing needles/syringes and marginally associated with initiating injecting outside Afghanistan (Table 2). HCV Ab was independently associated with initiating injecting outside Afghanistan, ever having an abscess, ever sharing needles and syringes, frequency of daily injection, and duration of injecting use in multivariable logistic regression (Table 2). HBV infection was independently positively associated with daily income and negatively associated with current needle and syringe program (NSP) use (Table 2).

**Table 2:** Variables independently associated with HIV and hepatitis B and C infection in logistic regression analysis among a cohort of male injecting drug users in Kabul, Afghanistan (N = 483)

Variable	Adjusted Odds Ratio	95% CI
HIV:		

Ever share needles or syringes	5.96	1.58 - 22.38

Initiated injecting outside Afghanistan*	3.41	0.83 - 14.27

Hepatitis B Virus:		

Daily income	1.01	1.00 - 1.01

Current needle and syringe program use	0.36	0.14 - 0.94

Hepatitis C Virus:		

Ever have abscess at injecting site	2.22	1.33 - 3.70

Ever share needles or syringes	2.33	1.38 - 3.95

Initiated injecting outside Afghanistan	1.95	1.26 - 3.04

Frequency daily injections (# injections)	1.47	1.11 - 1.94

Duration of injecting (per year)	1.05	1.00 - 1.10

Age (per year)	1.04	1.01 - 1.07

*Marginal significance (p = 0.087)

Most parsimonious models displayed.

CI = Confidence Interval

N = Number

### Prevalence and Correlates of NSP Service Use

Approximately half (53.8%) of the participants were using harm reduction services at time of enrollment and 51.3% reported receiving syringes from such a program in the last three months. HIV (1.9 vs. 2.3%, p = 0.82) and HCV (35.0% vs. 37.4%, p = 0.59) prevalence among NSP users was not significantly different than for non-users; age, civil status, educational level, and being homeless were also not significantly different between the groups. NSP use at enrollment was associated with prior incarceration, initiating drug use with injecting, frequency of daily injection, sharing of injecting works in the prior three months, and marginally with being homeless and receipt of prior drug treatment (Table 3). Living outside the country in the last five years and perceiving an urgent need for treatment were negatively associated with NSP use at enrollment in univariable logistic regression (Table 3). In multivariable logistic regression, initiating drug use with injecting, sharing injecting works in the past 3 months, prior incarceration, and greater frequency of daily injecting were independently associated with NSP use. Negative associations persisted for having lived outside the country in the last five years and a perceived great need for treatment (Table 3).

**Table 3:** Variables associated with needle and syringe program (NSP) use at enrollment among male IDUs in Kabul, Afghanistan in univariable and multivariable logistic regression analysis (N = 483)

	NSP Users (N = 258)	NSP Non-Users (N = 222)	
**Variable**	**Mean, SD**	**Mean, SD**	**OR, 95% CI**

Frequency of daily injection	5.78+0.70	5.55+0.94	1.43, 1.12 - 1.82

	N, %	N, %	

Lived outside last 5 years	156, 60.5%	154, 69.7%	0.66, 0.45 - 0.97

Prior incarceration	174, 67.4%	128, 57.9%	1.51, 1.04 - 2.19

Share injecting works last 3 mos	83, 32.2%	49, 22.1%	1.67, 1.11 - 2.53

Initiate drug use with injecting	28, 10.9%	11, 5.0%	2.34, 1.14 - 4.83

Perceived need for addiction therapy	243, 94.9%	219, 99.1%	0.17, 0.04 - 0.76

Receipt of prior addiction treatment*	45, 17.4%	25, 11.3%	1.66, 0.98 - 2.82

Homeless at enrollment*	66, 25.6%	42, 18.9%	1.47, 0.95 - 2.28

Multivariable Model:	AOR		95% CI

Initiated drug use with injecting	2.58		1.22 - 5.44

Shared injecting works in last 3 months	1.79		1.16 - 2.77

Prior incarceration	1.57		1.06 - 2.32

Frequency of daily injection	1.40		1.08 - 1.82

Lived outside Afghanistan in the last 5 years	0.61		0.41 - 0.91

Perceived need for addiction treatment	0.15		0.03 - 0.70

AOR = Adjusted Odds Ratio

CI = confidence interval

N = number

OR = odds ratio

SD = Standard Deviation

*marginal significance (p = 0.057 - 0.082)

## Discussion

The HIV, HCV, and HBV prevalence detected among Kabul IDUs from 2007 to 2009 was not substantially different from prevalence estimates obtained in 2006 [7]. Though factors believed to predispose to a rapid epidemic, such as poverty, unemployment, and insecurity have been consistently present, the explosive increase in HIV prevalence noted between annual cross-sectional studies in Pakistan and other settings has not yet occurred [12-14].

The association between HIV and HCV and lifetime needle or syringe sharing is consistent with 2006 findings and potentially indicates a sustained level of risk behavior among the population. However, reported recent sharing of either needles/syringes or injecting works were not associated with either infection. The reported levels of lifetime and recent sharing among Kabul IDUs in 2006 were much higher, as 50.4% reported ever sharing needles or syringes and 31% reported sharing injecting equipment in the six months prior to enrollment [7,15]. We hypothesize that harm reduction service expansion and inclusion of field-based NSP services has both decreased sharing and increased HIV transmission knowledge. However, the large decrease in reported lifetime sharing may indicate also that sharing is becoming a stigmatized behavior and is subsequently under-reported. The national drug use survey from 2009 reported lifetime needle sharing of 87% among IDUs; this survey included rural areas and noted a concentration of injecting in the southern provinces [5]. Few harm reduction programs operate in these provinces, where the population is predominantly rural, limiting the ability to disseminate information on the dangers of needle sharing. Since needle and syringe sharing remained strongly associated with prevalent HIV and HCV, there remains room for improvement in terms of harm reduction expansion in Kabul.

Initiating injecting outside Afghanistan was also associated with both HIV and HCV infection, likely reflecting increased likelihood of transmission in neighboring countries with higher HCV and HIV prevalence (e.g. Iran, Pakistan) [13,16]. The 2005 Kabul study did not assess where drug use and injecting were initiated, though injecting was assumed to be a behavior acquired outside Afghanistan [17]. As Afghans living as refugees may not have the same access to harm reduction/NSP services in countries of refuge, sharing needles and syringes may have been more likely, as observed in Iran [17].

HCV was associated with history of injection site abscess, potentially reflecting rushed injecting which may be a proxy measure for sharing of equipment [18,19]. In formative work, IDUs stated that relative speed of administration and ability to conceal use, particularly from police, were possible reasons for initiating injecting [20]. This same rationale may exist for rushed injecting. Having had an abscess at the injection site within the last year was associated with HCV antibody among IDUs in the United Kingdom in a cross-sectional study, [21] but a similar association was not observed in a Canadian study [19].

HBV infection was not associated with sharing needles/syringes or injecting in prison, as in 2006, but was negatively associated with current NSP use. As HIV and HCV were not associated with NSP use, it seems less likely that this may be a proxy for fewer risky sharing behaviors. However, the recent expansion of NSPs in Kabul and the greater virulence and ease of transmission associated with HBV may provide an earlier sign of positive behavior change associated with NSP use; forthcoming incidence data will be assessed for impact of NSP use on HBV and other infection incidence over time.

There was higher reported lifetime use of harm reduction programs, specifically NSP services, than measured in the 2006 study. As data presented here and in 2006 are cross-sectional and utilized convenience sampling, some of this difference may be attributed to undersampling NSP users in 2006 or oversampling this group during cohort recruitment. However, an increased number of harm reduction programs and introduction of primary exchange services in the field, now the preferred method for NSP delivery, have debuted and may contribute to this change [22].

Current NSP users represent IDUs who, in many respects, are at greater risk for blood-borne infection due to initiating drug use with injecting, recent sharing of injecting equipment, and more frequent injecting daily. That IDUs using NSP services are more likely to have risky injecting practices has been noted in other settings, such as the United Kingdom and Canada [23-25]. The data indicate that these services are reaching their target clientele; however, service delivery in this setting is a work in progress and efficacy remains an open question. NSP users were substantially less likely to perceive great need for treatment, potentially indicating NSP use is an entrée to addiction treatment. This relationship has been established in other settings and several harm reduction programs in Kabul have incorporated abstinence-based treatment programs [26,27]. As opioid substitution treatment (OST) is scaled up, harm reduction programs will need to ensure that linkages remain in place to treatment [26-28]. Further, programs should incorporate a low threshold model consistent with harm reduction rather than an abstinence based approach which has unrealistic expectations regarding the recovery process. The negative association between NSP use and having lived outside the country in the last five years may represent difficulties experienced by recently-repatriated IDUs in accessing services of which they may not be aware.

This study has several limitations. Participants were enrolled over a lengthy period by convenience sampling and may not be representative of IDUs in Kabul. Further, no data were recorded on those ineligible or declining entry, potentially leading to under-representation of hidden or isolated groups. We elected not to use respondent-driven sampling or other chain-referral methods in this setting for two reasons. First, based on qualitative work preceding cohort enrollment in Kabul, drug users perceive that injection drug use results in better services and greater opportunities for compensation [20]. Memorably, we were informed by one participant that press photographers would pay IDUs to be photographed or videotaped while injecting. By choosing and compensating seeds and recruits, we were concerned that drug users might inadvertently initiate injecting due to compensation, which may weigh on perceived ability to meet basic needs in this impoverished setting [29]. Similar concerns have been voiced by researchers in Lebanon [30]. We did not monetarily compensate our participants and also ensured that medical care, including VCT services and naloxone, were available to non-participants upon request. Next, in insecure environments where frequent migration outside and within the city occurs, networks are transient and assignment of seeds may not guarantee recruit derivation from the same network. This network fragility may then prompt "seeds" to recruit IDUs unknown to them, compromising the validity of RDS and increasing risk for coercive practices between seed and recruiter.

Socially desirable response may have occurred, particularly with sensitive behaviors; we attempted to reduce this through staff familiar to the participant population and providing choice as to location of the study interview. Though audio computer-assisted survey interviewing has been noted to improve self-reporting among IDUs, this technology was not financially possible or feasible in this environment where there was often no power and the team was largely field-based [31]. HIV-related analyses were underpowered due to low prevalence, potentially masking some associations. There were no female IDUs enrolled, precluding characterization. Female IDUs exist in Kabul but access to this hidden group has been elusive as the few women using harm reduction services are not injectors and require home visits due to cultural proscriptions.

## Conclusions

In summary, both injecting drug use and NSP utilization appear to be increasing in Kabul, Afghanistan. Injecting has become an accepted and popular route of drug administration, tied to many factors, with the ready availability of heroin among the most important. The apparent stability of HIV and HCV prevalence between an earlier study and this cohort at baseline may be offset by influx of new injectors and environmental factors favoring an explosive epidemic. Harm reduction program and NSP use appear acceptable and efforts to improve service quality and scale up effective interventions, particularly OST, are urgently needed.

## Abbreviations

The following abbreviations are used within the manuscript text: HBsAg: Hepatitis B surface antigen; HCV: Hepatitis C virus; HCV Ab: Hepatitis C antibody; HIV: Human immunodeficiency virus; IDU: Injecting drug user; NSP: Needle and syringe distribution and collection program; OST: Opioid substitution treatment; PCR: polymerase chain reaction; RIBA: Recombinant immunoblot assay; RPR: Rapid plasma regain; TPPA: Treponema pallidum plasma agglutination.

## Competing interests

The authors declare that they have no competing interests.

## Authors' contributions

CST designed the study, performed the data analysis, and drafted the manuscript. AN and MRS supervised data collection activities; AN also performed confirmatory testing procedures. KF performed quality assurance of field activities and supervised data entry. DV assisted with data analysis and interpretation and manuscript preparation. SAS assisted with study design, data interpretation, and manuscript preparation. All authors read and approved the final manuscript.
